# Case of Epilepsy of Two Years Standing Cured

**Published:** 1853-11

**Authors:** Geo. Fletcher

**Affiliations:** Hudson, Indiana


ART. III.—*Case of Epilepsy oj two years standing cured.* By Dr. Geo. Fletcher, Hudson, Inuiana.

Mrs. J., aged about 60 years, of very spare habits, tall and slim, was taken with fever and ague. This run on till bilious fever set in, and being reduced very low, I was sent for. I found her with considerable fever, and very weak, unable to rise from her bed, with pulse scarcely perceptible, tongue somewhat furred and considerable thirst. At the same time she complained of considerable weight in her stomach, as though a ball as large as her fist was there. Making some inquiries I learned that she was subject to fits, returning with exact regularity once in two weeks, and occuring on Saturdays and Sundays. These she had for two years.

Stimulants of small quantity with an active cathartic, followed by quinine, broke the fever.

During one of my visits she was taken with a fit. It was quite severe. It came on suddenly, with no premonition of its occurrence. Rigid spasms of the face and muscles occurred. The head was drawn to one side by short violent jerks, and the contraction of the muscles of the face distorted her features. Foaming of the mouth ensued, with violent beating of the breast with her fist.—This was followed by a dull comatose state for ten or fifteen minutes, when she was apparently as well as before.

During the fit, after the spasms had partly ceased, she vomited. This strained her a great deal, and she appeared as if something choked her. She afterwards told me she felt something in her throat which strangled her. The symptoms of worms was very plain, and I told her at once I thought her fits could be cured. I have been thus particular in detailing the symptoms of this case, that the grounds of my diagnosis and prognosis may be plainly seen.

As she had some fever at times, with slightly coated tongue, I directed her to take the following:

℞ Calomel, grs. 15
Pulv. Rhei., " 6

divided into three powders, and one taken every four hours. This was to be followed by:

℞ Castor Oil,
Ven. Turpentine, a a ℥ij.

This produced a free operation, but no discharge of worms. I now directed her to take of the following powders a large teaspoon full three times a day:

Sage pulv. ℥ss

Mustard ground ℥ij.

Ginger pulv. ℥jss m. ft. pulv.

This was continued nearly two weeks. During the administration of this, she took each day sufficient castor oil and ven. turpentine to produce a gentle catharsis. Shortly after taking the above powder she commenced voiding worms, from one to three or four daily, and in all discharged over two hundred of the ascarides lumbricoides. Since that time she has had no return of the fits, and has enjoyed good health The above powder seems to have antispasmodic as well as vermifuge properties, which adapt it to cases of epilepsy caused by worms.

